# Structure of intact human MCU supercomplex with the auxiliary MICU subunits

**DOI:** 10.1007/s13238-020-00776-w

**Published:** 2020-08-30

**Authors:** Wei Zhuo, Heng Zhou, Runyu Guo, Jingbo Yi, Laixing Zhang, Lei Yu, Yinqiang Sui, Wenwen Zeng, Peiyi Wang, Maojun Yang

**Affiliations:** 1 Ministry of Education Key Laboratory of Protein Science, Tsinghua-Peking Joint Center for Life Sciences, Beijing Advanced Innovation Center for Structural Biology, School of Life Sciences, Tsinghua University, Beijing, 100084 China; 2 Institute for Immunology and School of Medicine, Tsinghua-Peking Center for Life Sciences, Tsinghua University, Beijing, 100084 China; 3 Beijing Key Laboratory for Immunological Research on Chronic Diseases, Tsinghua University, Beijing, 100084 China; 4 Cryo-EM Facility Center, Southern University of Science & Technology, Shenzhen, 518055 China

**Dear Editor,**

Mitochondrial Ca^2+^ homeostasis regulates energy production, cell division, and cell death. The basic properties of mitochondrial Ca^2+^ uptake have been firmly established. The Ca^2+^ influx is mediated by MCU, driven by membrane potential and using a uniporter mechanism (Vasington and Murphy, 1962). Patch-clamp analysis of MCU currents demonstrated that MCU is a channel with exceptionally high Ca^2+^ selectivity (Kirichok et al., 2004).

Even in the absence of structural data on the MCU complex, mitochondrial Ca^2+^ uptake and its regulation in mammals has been assumed to rely on a complex comprising MCU, EMRE, MICU1, and MICU2 (De Stefani et al., 2016). Previous models generally believe that MICU1 and MICU2 form a cap to occlude the MCU channel in low [Ca^2+^] condition, and when [Ca^2+^] is elevated, through conformational changes of the EF hands in these two regulators, they will depart from the MCU/EMRE pore to allow Ca^2+^ permeation (Phillips et al., 2019).

In the present study, we tried to express the MCU complex in HEK 293F cells which were transfected by BacMam viruses for the genes *mcu*, *mcub*, *micu1*, *micu2*, and *emre*. After extensive optimization, we obtained an abundant amount of high quality human MCU-EMRE-MICU1-MICU2 supercomplex (MEMMS) protein samples, pulled-down by the C-terminally Strep-tagged EMRE. These samples were used to prepare grids for cryo-EM analyses (Fig. S1A, S1B and Methods). Images were recorded with a combination of a Titan Krios Cryo-EM and a K2 direct electron detector in super-resolution mode (Fig. S1C and S1D). After routine classification and refinement, further focused refinements for three regions were performed, and the three focused density maps were combined to generate an overall map of MEMMS at an improved resolution of 3.3–3.7 Å (gold-standard FSC 0.143 criterion) (Fig. S2). Compared to a most recent study (Fan et al., 2020), our MEMMS structure showed more accurate information about interactions between MICU1 and EMRE. Based on our structural and functional analysis, we conclude that MEMMS is an integral unit in mammals, EMRE may act as a lever to regulate the matrix gate of the MCU channel, and MICU1/2 enhance the Ca^2+^ uptake by interactions with the C- termini of EMRE in high [Ca^2+^] condition.

MEMMS has a molecular weight of about 480 kDa and an overall dimension of 210 Å × 190 Å. The overall structure forms an O-shaped ring and adopts the shape like that of two “goldfish”, as if glued together at both their heads (MICU1/MICU2 dimer) and tails (NTD of MCU). A pair of MICU1-MICU2 heterodimer appears like a bridge across the gap between the two halves of MCU-EMRE complex (Fig. 1A). The well-resolved density map allowed us to build a structural model for almost all residues with their side chains (Figs. 1A, S3, and S4). However, three sets of densities were not optimal for model building. The first is the density for the highly conserved C-terminal poly-D tail (EMRE^101−107^: EDDDDDD) of EMRE, the second is the conserved N-terminal poly-K (MICU1^99−102^: KKKK) of MICU1, and the third is the density for the conserved C-terminal helix (around 450–470) of MICU1 (Fig. S5A and S5B). The ambiguity of these densities might be owing to the double Strep tag we added, and the flexibility of these regions. Within the MEMMS structure, we modelled 2 Ca^2+^ ions at the E264 sites (MCU has high affinity for Ca^2+^ (≤2 nmol/L), E264 site is constitutively bound to Ca^2+^ under physiological conditions) (Kirichok et al., 2004), 8 cardiolipins (CDLs), and 16 phosphatidylcholines (PCs) (Figs. 1A, S3B and S3C). There is no direct interaction between MICU1 and MCU (Fig. 1A). When MICU1-FLAG plasmid was transfected into MICU2 KO HEK 293T cells, MICU1-FLAG was still able to co-precipitate with MCU, indicating that MICU2 is not required for interactions between MICU1 and MCU, which is consistent with our structure (Fig. 1B).

**Figure 1:**
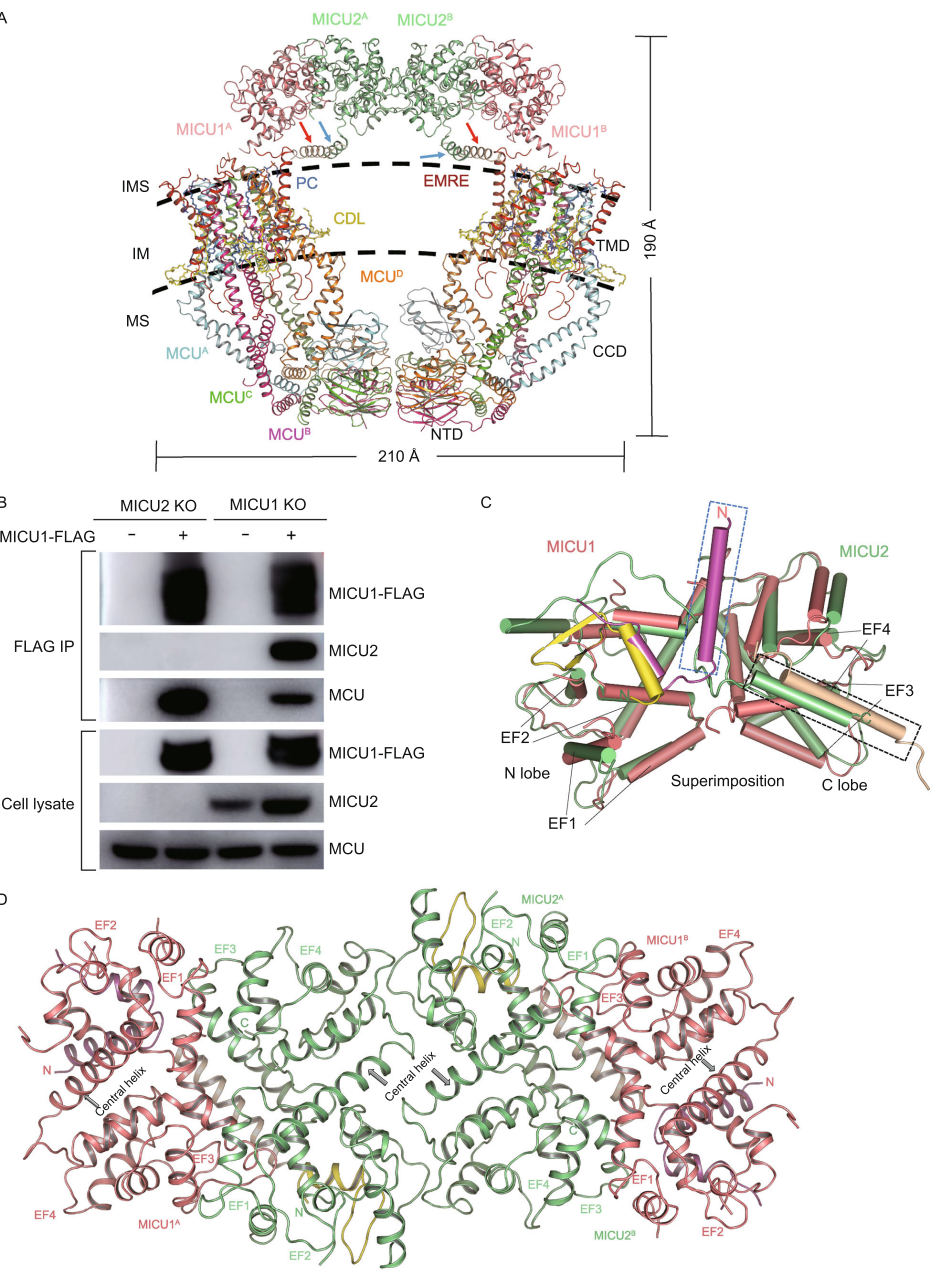

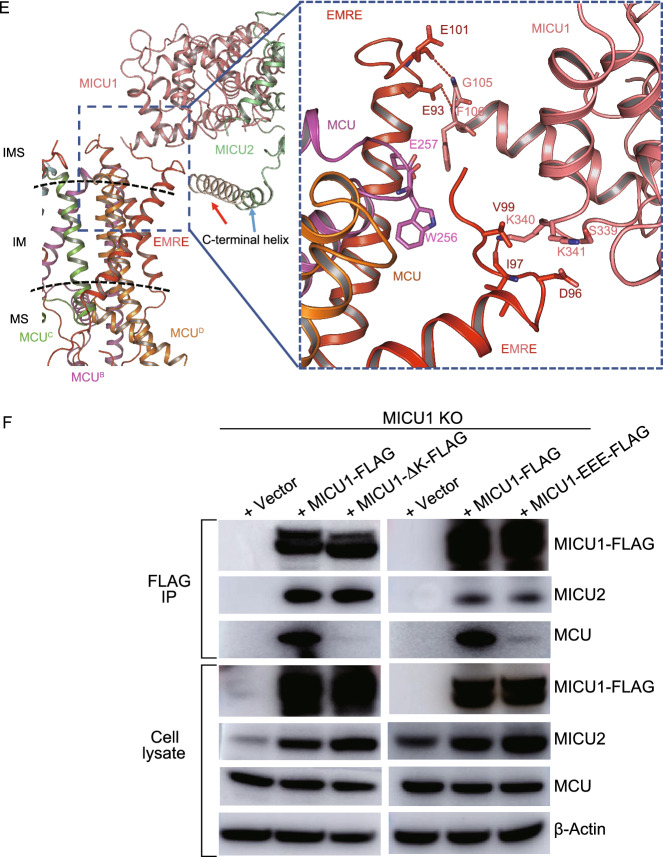
**Structure and validation of the MCU-EMRE-MICU1-MICU2 supercomplex.** (A) Atomic model of MEMMS. The position of NTD, CCD and TMD of MCU are indicated. PCs, phosphatidylcholines, are shown in marine; CDLs, cardiolipins, are shown in yellow; subunits of MEMMS are differently colored. IMS, intermembrane space; IM, inner membrane; MS, matrix. (B) FLAG co-immunoprecipitation of MICU1-FLAG expressed in MICU1 KO, MICU2 KO HEK 293T cells with transient expression of MICU1-FLAG. Lysates and elutes were immunoblotted with anti-FLAG, MCU or MICU2. (C) Cartoon representation and superimposition of the overall structure of MICU1 (the N-domain colored in magenta, the main body colored in light-pink and C-terminal helix colored in wheat) and MICU2 (the N-domain colored in yellow, the main body colored in light-green). The N-lobe, C-lobe, EF hand of each protein are indicated. The blue dashed box indicates the unique N-terminal helix of MICU1, the black dashed box indicates the C-terminal helix of MICU1. (D) The “face-to-face” interaction between MICU1-MICU2 heterodimers and the “back-to-back” interaction between two MICU2s. The colors are the same as in (C), the central helix of each subunit is labeled. (E) Interactions between EMREs and one MICU1. The blue dashed box indicates the interactions between the tails of two EMRE and the one MICU1, the right enlarged dashed box shows the detail. Residues responsible for interactions are shown as sticks. Hydrogen bonds are shown as red dashed lines. (F) FLAG co-immunoprecipitation of MICU1-FLAG and related mutant constructs expressed in MICU1 KO HEK 293T cells. Cells were transfected with MICU1-FLAG, MICU1 ΔK-FLAG or MICU1-S339E/K340E/K341E-FLAG plasmids (MICU1-EEE-FLAG). Lysates and elutes were immunoblotted with anti-FLAG, MCU, MICU2 or β-actin. Mean ± SEM, *n* ≥ 3

The MCU subunit comprises three structural domains: the transmembrane domain (TMD), the coiled-coil domain (CCD), and the N-terminal domain (NTD) (Fig. 1A). The TMD of MCU is known to be responsible for Ca^2+^ selectivity and conduction, and each MCU subunit contributes two transmembrane helices to the TMD: TM1 and TM2. Together, the four TM2s, which contain the highly conserved signature sequence (WDIMEP), form the inner wall of the Ca^2+^ channel, and the four TM1s form the exterior wall of the channel. On the IMS side, TM1 and TM2 are linked by a short loop, forming a hairpin structure with an opening angle of about 25° (Fig. S6A). An obvious gap is formed between the two helices on the matrix side, which is filled by one PC and one CDL molecules (Figs. 1A and S6A).

The CCD and NTD of MCU are located in the mitochondrial matrix. The CCD of each MCU subunit comprises three α-helices: an exceptionally long and obviously bent helix (CC1), a lateral helix (CC2), and a short helix (CC3). Helix CC1 is extended from TM1, forming the coiled-coil structure in CCD with helix CC3. Helix CC2 links TM2 and CC3 (Fig. S6A). The NTDs of the four MCU subunits align in a configuration resembling that of bent “goldfish tails”. The NTD is connected to the CC1 via the linking helix α1, and the four α1 helices of each MCU subunits stably interact with each other, forming a four-helix bundle that stabilizes the MCU tetramers (Fig. S6B).

MEMMS are linked at the IMS side via MICU1 and MICU2. Each MICU1 and MICU2 subunit contains four EF-hands, of which two are capable of binding Ca^2+^. Alignment of MICU1 and MICU2 shows that they have very similar core structures (N-lobe and C-lobe), but their N-terminal domains and C-terminal helices are different (Fig. 1C). MICU1 and MICU2 form a heterodimer in a previously reported “face-to-face” pattern, while the two MICU2 subunits interact in a ‘back-to-back’ pattern. Consequently, the N and C lobes of MICU1 and MICU2 subunits are arranged in an alternative pattern to link two MCU channels (Fig. 1C and 1D).

The C-terminal helices of both MICU1 and MICU2 also contribute to MICU localization onto the inner membrane (Fig. S7). In the MEMMS structure, although it’s difficult to analyze the detailed interactions between these two helices due to the vague local density, one can still appreciate that the two helices are parallel to each other at the surface of inner mitochondrial membrane (Figs. 1E and S3F). The C-terminal helix of MICU2 has hydrophobic residues partially buried in the inner membrane, while the positively charged residues point parallel to the membrane, interacting with the negatively charged phosphates of the membrane (Fig. S7). This is in agreement with the previous reports that MICU1 and MICU2 directly interact with the lipid membrane (Perocchi et al., 2010; Csordas et al., 2013). Previous reports also show that the C-terminal helix is important for the interaction of MICU1 with MCU complex. Accordingly, deletion of MICU1 C-terminal helix significantly weakened the binding of MICU1 to MCU, and even lowered Ca^2+^ uptake activity (Kamer and Mootha, 2014; Wang et al., 2014). In a previous study, Co-IP assay showed that MICU2 ΔC could not interact with MICU1 or MCU (Hoffman et al., 2013; Tsai et al., 2016). These findings are consistent with the MEMMS structure, in which the C-terminal helices act as an anchor to maintain MICU1 and MICU2 near to each other at the surface of inner mitochondrial membrane.

The negatively charged C-terminal loops of EMRE protrude into the IMS and are responsible for direct interaction with MICU1. The positively charged N-terminal poly-K (99–102) region of MICU1 and the negatively charged C-terminal tail (93–107) of EMRE are in close proximity to each other, as shown by clear interactions between MICU1 G105, F106 and EMRE E93, E101 (Fig. 1E), consistent with a previous functional study which has detected interactions between these two oppositely charged tails (Hoffman et al., 2013; Tsai et al., 2016). We deleted MICU1 poly-K region found that MICU1-ΔK cannot co-precipitate with MCU (Fig. 1F). In addition, we found an SKK (339–341) sequence in MICU1 C-lobe that can also interact with the negatively charged tail of another adjacent EMRE (Fig. 1E). So, we introduced a triple mutation (S339E, K340E, K341E) in MICU1 and found that the triple mutant also has reduced interaction with MCU (Fig. 1F). To conclude, the N-terminal domain and SKK sequence of MICU1 are important for its recruitment onto MCU/EMRE complex through interactions with the C-terminal tails of two adjacent EMRE subunits.

In our structure, four EMREs, four CDLs, four horizontal PCs, and four vertical PCs form a cage and bundle up the four MCU subunits (Fig. 2A and 2B). The N-terminal (48–65) and C-terminal (97–107) residues of EMRE appear as loops, while its middle residues (66–96) adopt the configuration of a single α-helix. This α-helix locates within the membrane and is tilted by 37° relative to the normal vector of membrane, such that each EMRE subunit interacts with two neighboring MCU subunits (Fig. 2B and 2C). The N-terminal loop of EMRE protrudes into the particularly large chamber of CCD, forming rich hydrogen bonds with CC2 and CC3, and even with a CDL molecule (Fig. 2C).

**Figure 2:**
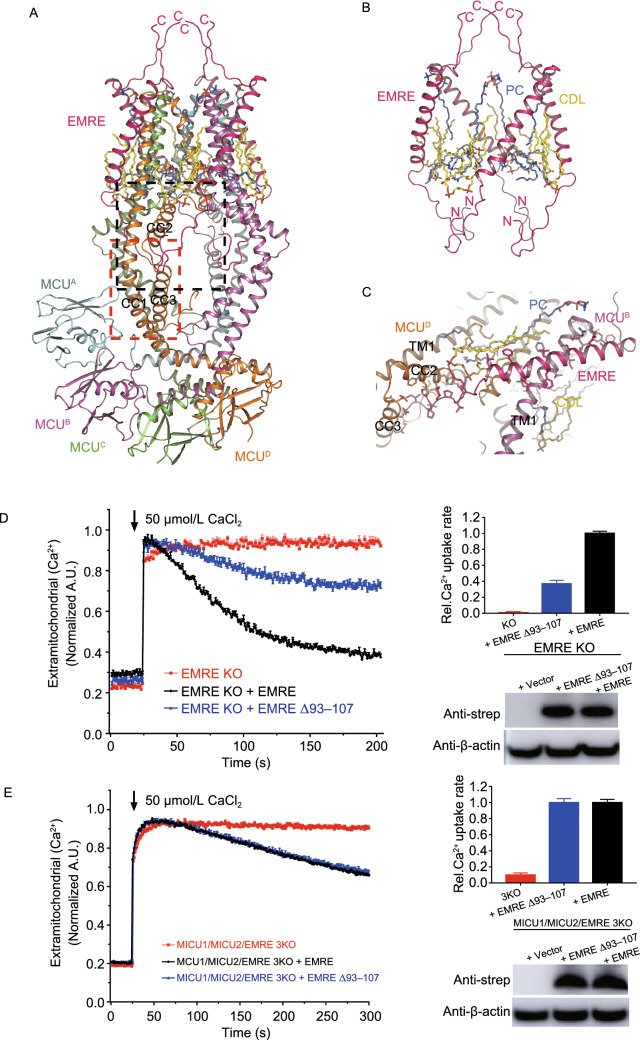

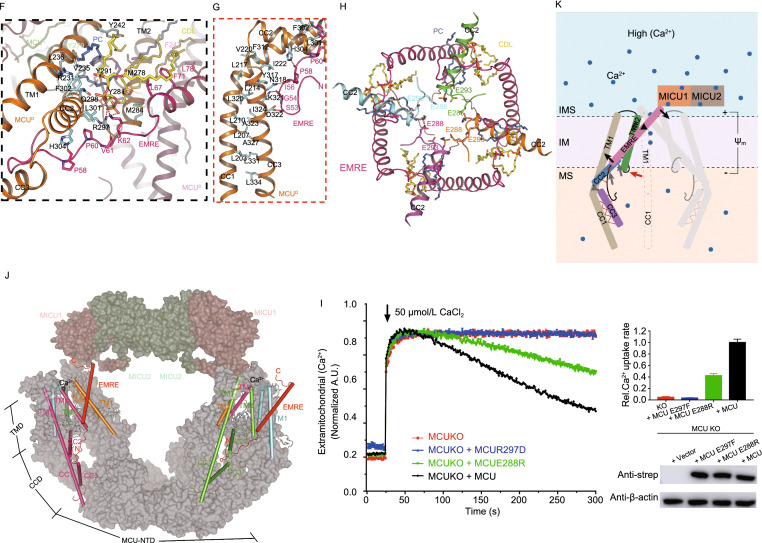
**Interactions, functional roles and a proposed regulation model of MEMMS**. (A) Interactions and functional roles of EMRE. Four EMRE subunits form a cage surrounding the TMD of MCU. MCU subunits are shown in different colors. Four EMRE subunits are distinguished by hotpink. Detailed interactions in colored dashed box are shown in (F and G), respectively. (B) EMRE subunits and phospholipids form a cage that bundles up the central MCU tetramer. All N and C-terminals of EMREs are noted. (C) Detailed interaction between an EMRE subunit and two MCU subunits. Transmembrane helix of EMRE interacts with TM1 of one MCU subunit, the N-terminal domain of EMRE interacts with the neighboring MCU CC2 and CC3. Residues responsible for interactions are labeled and shown as sticks. Hydrogen bonds are shown as red dashed lines. (D) The mitochondrial Ca^2+^ uptake of EMRE mutants at EMRE C-terminal in EMRE KO cells. Representative traces are shown on the left and bar graph on the right (mean ± SEM, *n* ≥ 3). Western blot of cell lysates from the different groups were performed to make sure  the amounts of protein expression were constant. β-actin was used as the loading control. (E) The mitochondrial Ca^2+^ uptake of EMRE mutants at EMRE C-terminal in MICU1/MICU2/EMRE triple KO cells. (F) Detailed interactions within the matrix gate of MCU complex. The black dashed box indicates interactions between CDL and surrounding subunits, including two MCU subunits and one EMRE subunit, and interactions between the N-terminal of EMRE and CC2 of MCU. (G) The red dashed box indicates the stable hydrophobic interface between MCU CC1 and CC3. Residues responsible for interactions are shown as sticks. (H) Intrusion of the PCs, CDLs and MCU CC2s into the central Ca^2+^ channel. E288s and E293s on CC2 are shown as sticks. (I) Mitochondrial Ca^2+^ uptake phenotype of MCU E288R or R297D mutant in MCU KO cells. (J and K) Proposed model of how EMRE and MICU regulate the conductivity of MCU supercomplex. (J) EMRE anchors on the TM1 of an MCU, while the N-terminal interacts with the CC2 and CC3 of the neighboring MCU in the matrix, and the C-terminal interacts with MICU1 in the IMS, thus linking up MICU and MCU. All TM2 of MCU, typical TM1 and neighboring CCD domain are shown as cylindrical helices, the rest of MEMMS are shown as surface. (K) Proposed gating mechanism of MCU in high [Ca^2+^]. Two sets of imagined levers are shown. EMRE is the first lever, with its pivot on TM1, its C-terminal loop attached to MICU1, and its N-terminal loop attached to CCD. CC2 is the second lever, with its pivot on the loop linking CC2 and CC3, its N-terminal attached to TM2, and its R297 attached to EMRE. R297 functions as the point of contact between the first and the second levers. Pivot and movement of the first lever are indicated by black triangle and arrows, respectively. Pivot and movement of the second lever are indicated by gray triangle and arrows, respectively. The movement of TM2 is marked by a red arrow. Membrane and membrane potential are labeled

We separately transfected intact EMRE and ΔC-EMRE (truncated of the negatively charged tail 93–107) into both EMRE KO and MICU1/MICU2/EMRE triple KO cells, and test the Ca^2+^ uptake rate of MCU in high [Ca^2+^]. In EMRE KO cells, reintroduction of EMRE fully rescued Ca^2+^ uptake, but reintroduction ΔC-EMRE can only rescue less than half of the total activity (Fig. 2D). Interestingly, in MICU1/MICU2/EMRE triple KO cells, reintroduction of EMRE and ΔC-EMRE have almost the same low rescue rate (Fig. 2E). These results indicate that enhancement effect of EMRE depends on its C-terminal tail, but without MICU1/2 even full length EMRE cannot enhance MCU activity. So, the recruitment of MICU1/2 through EMRE C-termini is indispensable for enhancing MCU Ca^2+^ uptake in high [Ca^2+^].

In the MEMMS structure, one CDL and one horizontal PC molecules insert into the gap between TM1 and TM2 of each MCU subunit, and another vertical PC molecule stands alongside each TM2. Notably, most of the lipid chains of the CDL and vertical PC in our structure are parallel to the helices of TMD, while the lipid chains of the horizontal PC are positioned horizontally in the membrane (Figs. 2A, S3C and S6A). In addition to interacting with TM1, TM2, and CC2 of one MCU subunit, each CDL molecule also interacts tightly with the neighboring EMRE. Specifically, a highly conserved residue of MCU CC2, R297, can form salt bridge with the phosphate group of CDL and hydrogen bonds with the main chain oxygen of V61 in EMRE (Fig. 2F). We mutated R297 to aspartate, and strikingly this mutation completely abolished the Ca^2+^ uptake via MCU (Fig. 2I). Similarly, P60A mutation in EMRE, just next to the MCU-R297, can also totally abolish MCU activity (Yamamoto et al., 2016), proving that the correct interactions between CC2 and EMRE are important to MEMMS.

It has been proposed that CC2 and TM2 form a luminal gate near the matrix side of MCU that is maintained in an open conformation via its interaction with EMRE (Wang et al., 2019; Yamamoto et al., 2019). Both the previous human MCU-EMRE structure and our MEMMS structures have stable hydrogen bonds between EMRE N-terminal loop and MCU CC2-CC3 (Fig. 2F), however, we also found several phospholipids filling the gaps between helices from MCU and EMRE. These phospholipids could stabilize the gaps and provide elasticity to this region, enabling the gate to be opened by EMRE (Fig. 2H). The MCU-R297D mutation might dissociate the bound CDL and disrupt the attachment of EMRE on CC2. This would leave CC2 free to roll aside and possibly push the negatively charged E288 and E293 residues of MCU inward, thus making the channel non-conducting.

Furthermore, we found that MCU E288R mutation can also severely reduce the Ca^2+^ uptake activity, supporting the matrix gate to be indispensable for Ca^2+^ transport (Fig. 2I). We also observed multiple hydrophobic interactions between CC3 and CC1, which might help to achieve the correct position of the gate-forming CC2 (Fig. 2G). The amino acid residues participating in these hydrophobic interactions are highly conserved and were shown to be indispensable for MCU activity (Fig. S5C) (Yamamoto et al., 2019). Besides, the negatively charged phosphate group of the horizontal PC is also very likely involved in forming the gate, because their conformation is quite stable and they protrude deeply into the ion-conducting pore (Figs. 2H and S8C).

Garg et al. demonstrates that Ca^2+^ binding to MICU1/2 potentiates Ca^2+^ permeation through the MCU pore by increasing the probability of its open state (Vivek Garg et al., 2020). Our MEMMS structure and the previously proposed MCU gating mechanism (Wang et al., 2019) provide a reasonable explanation for the functional behavior of the MCU complex. We hypothesize that after Ca^2+^ binding to their EF-hands, a conformational change in MICU1/2 dimers exerts a force upon EMRE and the elastic MCU matrix gate, thus increasing its probability of open state. Specifically, a pair of MICU1/2 heterodimer links the V-shaped MCU-EMRE complex, making it possible to generate a pulling force on EMRE when their conformations are changed. EMRE could function as a lever, with its C-terminal loop interacting with MICU1, its central helix anchored to TM1 of MCU as the pivot, and its N-terminal loop supporting MCU CCD. The interaction between MCU R297 and EMRE V61 is the force bearing point of CC2. In addition, rich phospholipids around the MCU matrix gate could provide elasticity to this region, enabling the movement of neighboring helices. After Ca^2+^ binding, conformational changed MICU1/2 could push the EMRE N-terminal through the lever, dragging CC2 and TM2 away from the center of pore, and consequently cause the enlargement (or stabilization of the open state) of the MCU matrix gate (Fig. 2J and 2K).

Furthermore, we compared our MEMMS structure with previous fungal MCU structures to find that human MCU has a swollen CCD enlarged by EMRE (Fig. S8A–C). The curvature of CC1 is very likely facilitated by P216, which is conserved in mammals but absent in fungi (Fig. S8D). The CC2s in reported fungal MCU structures are not well resolved, indicating that their position is flexible possibly due to lack of EMRE. The curvature of CC1 and the tight CC1-CC3 interaction in human MCU could probably elevate the position of CC2 and close the gate if no EMRE is bound. Consequently, we propose that because fungal MCU does not have an elevated CC2, it does not require EMRE to maintain an open position. In contrast, EMRE is indispensable for human MCU because its CC2 is supported by EMRE N-terminal loop.

In conclusion, here we report the structure of intact MCU supercomplex as a 20-subunit O-shaped dimer of hetero-decamers, with auxiliary MICU1 and MICU2 subunits attached. We found that MICU1 does not directly contact MCU, but can attach onto the MCU complex through interaction with EMRE, indicating that a critical function of EMRE is to couple the Ca^2+^ sensing MICUs with the MCU activity. We propose that the recruitment of MICU1/2 through EMRE C-termini exert a pulling force upon EMRE lever to stabilize the open state of the MCU matrix gate.

## Electronic supplementary material

Below is the link to the electronic supplementary material.

Supplementary material 1 (PDF 111146 kb)
