# Supplementary material for: Structure of intact human MCU supercomplex with the auxiliary MICU subunits
Source: Protein Cell. 2020 Aug 30;12(3):220–9. doi: 10.1007/s13238-020-00776-w (PMC7895871; doi:10.1007/s13238-020-00776-w)
Supplement: Supplementary file 1 — Supplementary material 1 (PDF 111146 kb) [file 13238_2020_776_MOESM1_ESM.pdf]

MATERIALS AND METHODS

Co-expression of human mitochondrial Ca^{2+} uniporter supercomplex

The optimized coding DNAs for *H.sapiens mcu* (Uniprot: Q8NE86), *mcub* (Uniprot: Q9NWR8), *micu1* (Uniprot: Q9BPX6), *micu2* (Uniprot: Q8IYU8) and *emre* (Uniprot: Q9H4I9) were synthesis in GenScript and cloned into the pEG BacMam vector (Goehring et al., 2014), with tandem twin Strep-tag or FLAG tag at the C terminus of these five proteins. The BacMam viruses were produced and amplified in Sf9 cells. After extensively biochemical studies, we obtained the high quality and quantity protein complexes with the combination of the EMRE C-terminus tagged and the other four subunits without tags viruses. For larger amount protein purification, four liters of the HEK 293F cells were cultured. When the cell density reached 2×10^6 cells per mL, the cells were transfected with the P3 (the third passage) BacMam viruses of MCU, MICU1, MICU2, MCUb and EMRE, each at 8 mL per liter cell culture. Transfected cells were cultured for 48 hours before harvesting.

Purification of the human mitochondrial Ca^{2+} uniporter supercomplex

All procedures were carried out at 4°C. To purify the MEMMS complex, four liters of transfected cells were harvested, washed with 1×PBS and resuspended in 100 mM Tris pH 7.4, 225 mM sorbitol, 60 mM KCl, 0.1 mM EGTA, and 0.1% BSA, 1 mM PMSF. The suspension was homogenized by a soft blender for 150 s and the homogenate was centrifuged at 3,000 *g* for 10 min. The supernatant was further centrifuged at 20,000 *g* for 45 min to obtain the crude mitochondria. The pellet was suspended and extracted in 25 mM Tris pH 7.8, 150 mM NaCl, 0.1 mM EGTA with 1% digitonin. After incubation for an hour, the extraction was centrifuged at 20,000 *g* for 20 min at 4°C and the supernatant was applied to Strep-Tactin Sepharose by gravity at 4°C. The resin was washed three times with W buffer, which contained 25 mM Tris pH 7.8, 150 mM NaCl, 0.1 mM EGTA with 0.1% digitonin. The target proteins were eluted with W buffer plus 5 mM desthiobiotin, concentrated to 100 μL by 100 kDa cut-off centrifugal filter (Millipore) and further purified by Superdex200 increase 5/150 GL also in W buffer. The peak fractions were collected for EM sample preparation, the presence of the complex was verified by Blue Native-PAGE and confirmed by mass spectrometry.

Sample preparation and cryo-EM data acquisition

4 μL aliquots of freshly purified MEMMS at a concentration of 5 mg/mL were placed on glow-discharged 400-mesh Quantifoil R1.2/1.3 grids (Quantifoil, Micro Tools GmbH, Germany). Grids were blotted for 5 s and flash-frozen in liquid ethane using an FEI Mark IV Vitrobot operated at 8°C and 100% humidity. The grids were transferred to a Titan Krios (FEI) electron microscope equipped with a Cs corrector, operating at a voltage of 300 kV. Images were recorded by a K2 Summit direct electron detector (Gatan, Inc.) equipped with a GIF Quantum energy filter (slit width 20 eV) in the super-resolution counting mode. Data acquisition was performed using AutoEMation II with a nominal magnification of 105,000 times, which yields a super-resolution pixel size of 0.5455 Å on the image plane, and with defocus ranging from -1.5 μm to -2.0 μm. The dose rate on the detector was ~8.0 counts per pixel per second with a frame exposure time of 0.175 second and a total exposure time of 5.6 seconds. Each micrograph stack contains 32 frames. The total dose rate was approximately 50 $e^-/Å^2$ for each micrograph.

Image processing

A simplified flowchart of the procedure for image processing of MEMMS is presented in Fig. S2. A total of 9,899 cryo-EM movie stacks were motion-corrected, 2×2 binned and dose weighted using MotionCor2. After whole image CTF estimation using CTFFIND3, 9,113 good micrographs were manually selected from the dataset. A total of 986,805 particles were autopicked using RELION-3.0. After several rounds of 2D classification, 699,244 particles were selected and subjected to 3D classification, using MCU-EMRE map (EMD-9944) low-pass filtered to 50 Å as the initial model. After 3D classification, 250,977 particles of the class with density of MICUs were selected then subjected to 3D refinement. Each particle was recentered and re-extracted from the motion-corrected integrated micrographs. Gctf was used to refine the local defocus parameters. The re-extracted particles were subjected to 3D refinement without symmetry, which resulted in a map at 4.09 Å resolution.

The 250,977 particles were further classified into three classes using masked skip-alignment 3D classification. A total of 45,864 particles of the class with clear density of MICUs were subjected to 3D refinement with a soft mask then yield a map at 3.64 Å resolution. Since the density of the MICUs (i.e. the “cap”) is still fragmentary, these particles were subtracted by the density of MCU-

EMRE in the 3.64 Å map of MEMMS using RELION-3.0 and subjected to 3D refinement. Then 33,930 particles were selected after a final round of 3D classification and subjected to 3D refinement with a soft mask and C2 symmetry, leading to a reconstruction of MICUs at 3.71 Å resolution with much better density. To improve the density of NTDs, the 45,864 particles were subtracted by the density of CCDs, TMDs and MICUs, then subjected to 3D refinement with a soft mask and C2 symmetry, resulting in a density map of NTDs at 3.39 Å resolution. To improve the density of TMD+CCD, the 45,864 particles were expanded according to C2 symmetry using relion_particle_symmetry_expand, subtracted by the density of the rest part beside TMD+CCD, then subjected to 3D refinement with a soft mask. Eventually, the resolution of TMD+CCD was improved to 3.30 Å. The focused map of NTDs, MICUs and two copies of the focused map of TMD+CCD were fit into the 3.64 Å map then combined using PHENIX Combine Focused Maps, resulting in the final map.

The reported resolutions are based on the gold-standard Fourier shell correlation 0.143 criterion. All density maps were sharpened by applying a negative B-factor that was estimated using automated procedures. Local resolution variations were estimated using Resmap.

Model building and refinement and validation

The atomic model of MCU and EMRE was manually built and adjusted in COOT (Emsley et al., 2010). And then, the model with the ligands was subjected to global refinement and minimization in real space refinement using PHENIX with secondary structure and NCS restraints. The crystal structure of MICU1 (PDB accession 4NSC) and MICU2 (PDB accession 6AGH) were used as the initial models for MICUs in MEMMS. The model was refined in real space using PHENIX with secondary structure and NCS restraints. The final atomic models were evaluated using MolProbity (Davis et al., 2007). Pore radii were calculated using the HOLE program (Smart et al., 1996). All the figures were prepared in PyMol (Alexander et al., 2011).

Gene knockout by CRISPR/Cas9

Gene knockout by CRISPR/Cas9 was performed using a previously described protocol (Ran et al., 2013). Two sets of guide RNA sequences were designed. Guide sequences used for gene knockout were as follows:

MCU-KO1: CAGGAGCGATCTACCTGCGG; MCU-KO2: TGAACTGACAGCGTTCACGC; EMRE-KO1: GGCTAGTATTGGCACCCGTC; EMRE-KO2: TACTAGCCAGCGAGCCGCTC; MICU1-KO1: AAACCAGTATGGGTATGCGC; MICU1-KO2: CGAATTTCAGCGTAAACTGC; MICU2-KO1: CAGCCGCGTCAGTGTTGCGG; MICU2-KO2: TGGGGCGGAAAACTGCGACG. After sequencing, HEK 293T cells were transfected transiently with pSpCas9(BB)-2A-GFP plasmid which contained the corresponding guide sequence. Single cell was isolated by flow cytometry 24 hours later and proliferated for 2 weeks. Gene knockout was confirmed by sequencing and western blot.

Co-immunoprecipitation and western blot

All co-immunoprecipitation experiments were performed at 4°C. In brief, related HEK 293T knockout cells at 80%-90% confluence were transfected with 15 μg corresponding plasmids using lipofectamine 2000 (Thermo Fisher Scientific) and grown in a 37℃ CO_2 incubator for 24 hours. Transfected cells were lysed in 1 mL lysis buffer (25 mM Tris pH 7.8, 150 mM NaCl, 1 mM EGTA, cOmplete protease inhibitors) with 1% digitonin. The cell lysate was incubated for 30 min on ice and centrifuged for 10 min at 4°C at 20000 *g*. A small portion of the sample was used for whole cell lysate analysis and the rest was collected and incubated with anti-FLAG magnetic agarose (Thermo Fisher Scientific) for two hours at 4℃. The beads were collected on a magnet, washed three times with 1 mL lysis buffer which contained 0.1% digitonin, and eluted with 150 μL SDS-gel loading buffer for western blot.

For western blot analysis, proteins were subjected to a 4-20% SDS-PAGE gel (GenScript) and transferred onto a PVDF membrane (Millipore). Membranes were detected with the indicated antibodies. The primary antibodies were used: MCU (Abcam), MICU1 (Sigma-Aldrich), MICU2 (Sigma-Aldrich), FLAG (Easybio), Strep (Easybio), Mouse-β-actin (Easybio).

Mitochondrial Ca^{2+} uptake assays

Mitochondrial Ca^{2+} uptake was performed on MCU KO ($MCU^{-/-}$), EMRE KO ($EMRE^{-/-}$), or MICU1/MICU2/EMRE triple KO ($MICU1^{-/-}/MICU2^{-/-}/EMRE^{-/-}$) HEK 293T following the published protocol (Kamer and Mootha, 2014). Briefly, the transfected cells were digested, washed with 10 mL PBS for three times and re-suspended in buffer (25 mM HEPES pH 7.4, 125 mM KCl, 2 mM

KH_2PO_4, 1 mM $MgCl_2$, 10 μM EGTA, 5 mM glutamate, 5 mM malate, 3 μM thapsigargin, 0.005% digitonin, 1 μM Oregon Green-Bapta6F) to a final concentration of 10×10^6 cells per mL. 150 μL cell suspension was transferred into a 96-well plate (Corning). Fluorescence was recorded using a Perkin/Elmer plate reader with excitation 488 nm/emission 535 nm before and after Ca^{2+} injection. 50 μM $CaCl_2$ was injected, resulting in about 40 μM free Ca^{2+}. The relative Ca^{2+} uptake rate is reported as the linear fit of the fluorescence for 3 or 5 minutes. To normalize the fluorescence readout, the maximal fluorescence value was set to 1.0 and the other fluorescence values at each time point were divided by the maximal fluorescence. The relative rate of Ca^{2+} uptake was analyzed in GraphPad Prism 7 (GraphPad Software, Inc.). Western blot was performed to ensure protein expression was comparable among the MCU or EMRE mutants in the uptake assay, Mouse anti-β-actin was used as a loading control.

REFERENCES

Alexander, N., Woetzel, N., and Meiler, J. (2011). bcl::Cluster : A method for clustering biological molecules coupled with visualization in the Pymol Molecular Graphics System. IEEE Int Conf Comput Adv Bio Med Sci 2011, 13-18.

Davis, I.W., Leaver-Fay, A., Chen, V.B., Block, J.N., Kapral, G.J., Wang, X., Murray, L.W., Arendall, W.B., 3rd, Snoeyink, J., Richardson, J.S., *et al.* (2007). MolProbity: all-atom contacts and structure validation for proteins and nucleic acids. Nucleic Acids Res 35, W375-383.

Emsley, P., Lohkamp, B., Scott, W.G., and Cowtan, K. (2010). Features and development of Coot. Acta Crystallogr D Biol Crystallogr 66, 486-501.

Goehring, A., Lee, C.H., Wang, K.H., Michel, J.C., Claxton, D.P., Baconguis, I., Althoff, T., Fischer, S., Garcia, K.C., and Gouaux, E. (2014). Screening and large-scale expression of membrane proteins in mammalian cells for structural studies. Nat Protoc 9, 2574-2585.

Kamer, K.J., and Mootha, V.K. (2014). MICU1 and MICU2 play nonredundant roles in the regulation of the mitochondrial calcium uniporter. EMBO Rep 15, 299-307.

Ran, F.A., Hsu, P.D., Wright, J., Agarwala, V., Scott, D.A., and Zhang, F. (2013). Genome engineering using the CRISPR-Cas9 system. Nat Protoc 8, 2281-2308.

Smart, O.S., Neduvelil, J.G., Wang, X., Wallace, B.A., and Sansom, M.S. (1996). HOLE: a program for the analysis of the pore dimensions of ion channel structural models. J Mol Graph 14, 354-360, 376.

Table S1. Cryo-EM data collection, refinement and validation statistics

	MCU-EMRE-MICU1-MICU2 supercomplex
**Data collection and processing**	
Magnification	105,000
Voltage (kV)	300
Electron exposure ($e^-/Å^2$)	50
Defocus range (μm)	-1.5 ~ -2.0
Pixel size (Å)	1.091
Software	RELION-3.0
Symmetry imposed	C2
Initial particle images (no.)	986,805
Final particles images (no.)	33,930 / 45,864 / 91,728*
Map resolution (Å)	3.71 / 3.39 / 3.30*
FSC threshold	0.143
Map sharpening *B* factor	-113 / -92 / -96*
Local map resolution range (Å)	5.0-3.5 / 4.5-3.0 / 4.5-3.0*
**Refinement**	
Software	PHENIX 1.15
Initial model used (PDB code)	4NSC / 6AGH
Model resolution (Å)	3.4
FSC threshold	0.5
Model composition	
Non-hydrogen atoms	32954
Protein residues	3872
Ligand	24
B factors ($Å^2$)	
Protein	55.37
Ligand	54.94
R.m.s deviations	
Bond length (Å)	0.003
Bond angles (°)	0.527
Validation	
MolProbity score	1.68
Clashscore	6.72
Poor rotamers (%)	0.09
Ramachandran plot	
Favored (%)	95.49
Allowed (%)	4.51
Disallowed (%)	0.00

* The values for the density map of MEMMS MICUs, NTDs and TMD+CCD, respectively.

Fig. S1. Structural characterization of MCU supercomplex.

A Representative size-exclusion chromatography profile of the MEMMS. **B** Protein samples of the size-exclusion chromatography fractions were subjected to BN-PAGE. Fractions of corresponded elution volume were used for cryo-EM sample preparation. **C** Representative micrograph of the MEMMS. **D** 2-D class averages for the cryo-EM structure of MEMMS.

Fig. S2. Flowchart of EM data processing and statistics of the final density map of MEMMS.

Details of data processing are described in the 'Image processing' section of the Methods.

Fig. S3. Representative density maps of MCU super complex.

A Interaction between EMRE and MCU. **B** The electron densities of PC and CDL. **C** Polar heads of PC intruding into central channel. **D** Interaction between two MCU tetramers through pairs of NTDs. **E** Entrance of central channel of MEMMS. The density of one Ca^{2+} ion is shown. **F** C-terminal helix electron densities of MICU1 and MICU2. **G** Representative electron densities of interactions between MICU1 and MICU2. **H** Representative electron densities of MICU2.

Fig. S4. Model building of the MCU supercomplex.

A Combined density map and structural model of MEMMS, model of MEMMS is colored brown. **B** Local resolution of the density map of MICU1 and MICU2 of MEMMS. Angular orientation distribution of the particles. **C** Local resolution of the density map of TMD and CCD of MEMMS. Angular orientation distribution of the particles. **D** Local resolution of the density map of NTD of MEMMS. Angular orientation distribution of the particles. **E** Gold-standard Fourier shell correlation (FSC) curve of the final density maps of MEMMS MICU1+MICU2 (blue), MEMMS TMD+CCD (orange), and MEMMS NTD (brown).

Fig. S5. Structure-based EMRE, MICU1 and MCU orthologues alignment.

A The amino acid sequences of *H. sapiens, B. taurus, M. musculus, X. tropicalis, D. melanogaster, D. rerio* and *C. elegans* EMRE are aligned and colored according to the ClustalW convention (Uniprot accession numbers: Q9H4I9, Q2M2S2, Q9DB10, Q28ED6, Q7JX57, A0A0J9YJ98 and Q9U3I4, respectively). Secondary structure represented by ribbons is based on the cryo-EM structure of *H. sapiens* EMRE. The conserved amino acid in the N terminal and the poly-aspartic tail are indicated. **B** The amino acid sequences of MICU1 are aligned and colored according to the ClustalW convention as in A. Secondary structure represented by ribbons is based on PDB 4NSC. The poly-lysine region and SKK region are indicated in the dashed box. **C** The amino acid sequences of MCU are aligned and colored according to the ClustalW convention as in A. Secondary structure represented by ribbons is based on the cryo-EM structure of *H. sapiens* MCU. TM1 and TM2 are conserved and indicated in the black dashed box. CC1, CC2 and CC3 domains are conserved and indicated in the green dashed box. The conserved Arg in CC2 is indicated.

Fig. S6. TMD, CCD and NTD of MCU.

A Overall structure of the MCU monomer. Membrane is indicated by two dashed lines. CDL and PC are shown as sticks, colored in yellow and marine respectively. MCU monomer is shown as cartoon and colored as rainbow. TM1 and TM2 form TMD. CC1, CC2, and CC3 form CCD. The linking helix α1 links CCD and NTD. **B** Linking site between CCDs and NTDs of MCU tetramer. Helices from four monomers are distinguished by different colors. C termini of different MCU monomers are indicated. Four α1s stack into two layers stabilizing the matrix region.

Fig. S7. The C-terminal helix of MICU is important for MCU complex function.

A Superimposition of MICU1 (colored in deep-salmon) in the MEMMS and hMICU1 in the Ca^{2+} free state (PDB: 4NSC, colored in marine) in different views. 4NSC C-terminal helix is circled in the

red dashed line. **B** Surface electrostatic potential analysis of 4NSC C-terminal helix. The helix is positively charged on one side and hydrophobic on the other side. Amino acid sequences of *H.sapiens, B. taurus, M.musculus, X.tropicalis,* and *D.rerio* MICU1 C-terminal helix are aligned according to the ClustalW (Uniprot accession numbers: Q9H4I9, Q2M2S2, Q9DB10, Q28ED6, and A0A0J9YJ98, respectively). Secondary structure represented by ribbons is based on the C-terminal helix structure of 4NSC. Conserved residues among species are highlighted in red. **C** Superimposition of MICU2 (colored in lime-green) in the MEMMS and hMICU2 in the Ca^{2+} free state (PDB: 6EAZ, colored in violet) in different views. 6EAZ C-terminal helix is circled in blue dashed line. **D** Surface electrostatic potential analysis of 6EAZ C-terminal helix. The helix is positively charged on one side and hydrophobic on the other side. Amino acid sequences of MICU2 C-terminal helix are aligned as in B. Secondary structure represented by ribbons is based on the C-terminal helix structure of 6EAZ. Conserved residues among species are highlighted in red.

Fig. S8. Comparison between different MCU channel.

A Superimposition of four fungal MCU structures (PDB 6D7W colored limon, 6C5W colored purple, 6DNF colored orange, and 6DT0 colored hot-pink). TM1 and TM2 in the transmembrane domain (TMD), CC1, CC2 and CC3 in CCD, and NTD domain are indicated. The black dashed box shows that the interactions in CCD are conserved. The enlarged black dashed box shows the hydrophobic interactions between CC1 and CC3. **B** Alignment of *N. fischeri* MCU TMD+CCD domain (PDB 6D7W, colored limon) and *H.sapiens* MCU TMD+CCD domain (colored salmon) based on TMD. TM1, TM2, CC1, CC2, and CC3 helixes are indicated. The salmon arrows indicate the rotate directions of *H.sapiens* CC1, CC2, compared to *N. fischeri* CC1, CC2, respectively. Pro216 in *H.sapiens* CC1 is shown in sticks. **C** Pore radius along the ion conduction pathway of the indicated MCU structures. The gate residues and PC are labeled. MEMMS colored salmon, *H.sapiens* MCU+EMRE (PDB 6O58) colored in magenta, *N. fischeri* MCU (PDB 6D7W) colored limon, and *H.sapiens* MCU+EMRE+MICU1+MICU2 (PDB 6WDO) colored purple-blue. **D** The amino acid sequences of *H. sapiens, B. taurus, M. musculus, N. fischeri, C. europae , M. acridum* and *N. crassa* TMD+CCD are aligned and colored according to the ClustalW convention (Uniprot accession numbers: Q9H4I9, Q2M2S2, Q9DB10, A1CWT6, W2SDE2, E9DVV4 and Q7S4I4, respectively). Secondary structure represented by ribbons is based on the cryo-EM structure of *H. sapiens* TMD+CCD. The blue and green dashed boxes indicate the conserved Pro in CC1 and Arg in CC2 of higher eukaryotes, respectively.

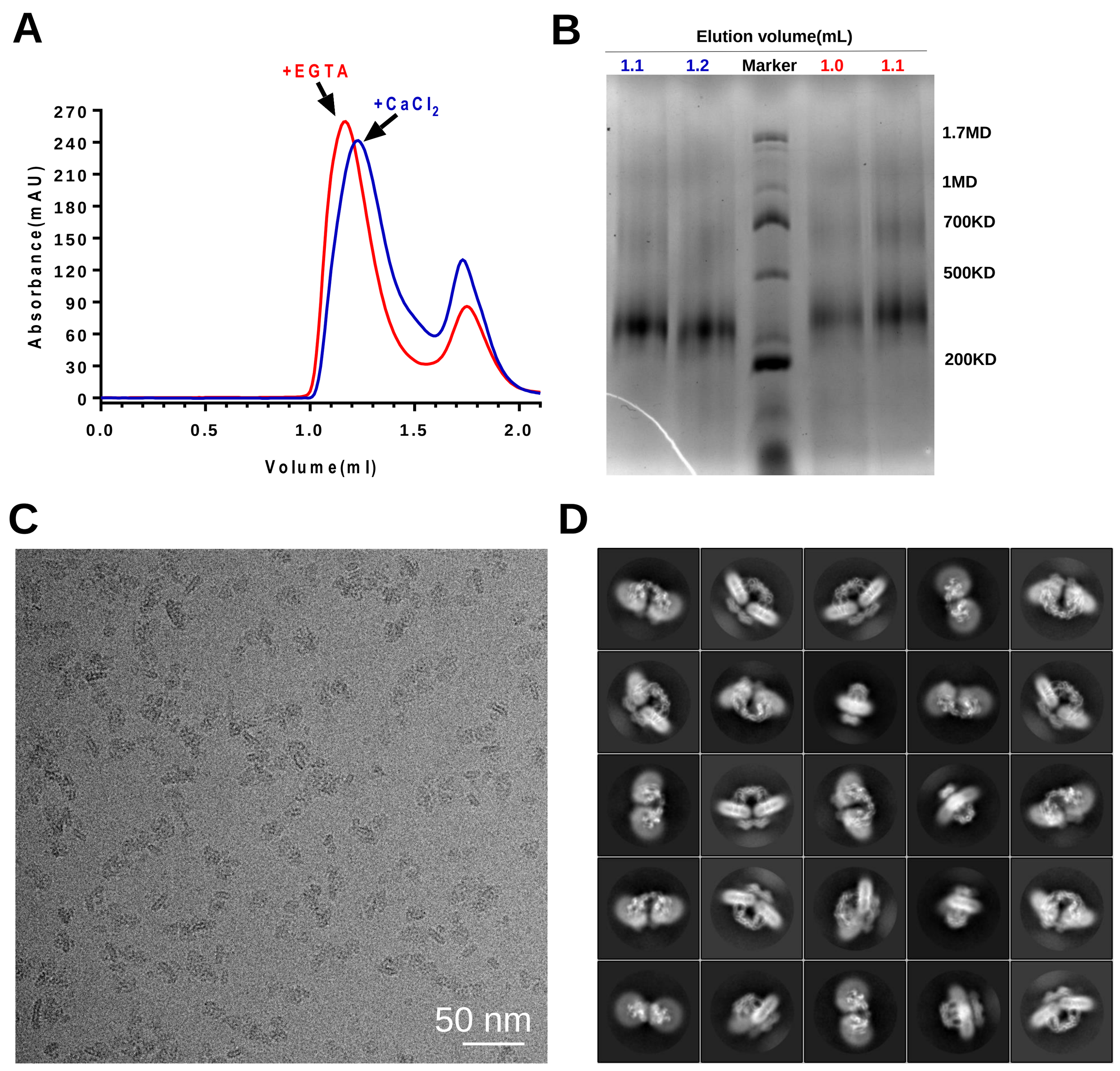

A
+EGTA
+CaCl2
Absorbance(mAU)
270
240
210
180
150
120
90
60
30
0
0.0
0.5
1.0
1.5
2.0
Volume(ml)
B
Elution volume(mL)
1.1
1.2
Marker
1.0
1.1
1.7MD
1MD
700KD
500KD
200KD
C
50 nm
D

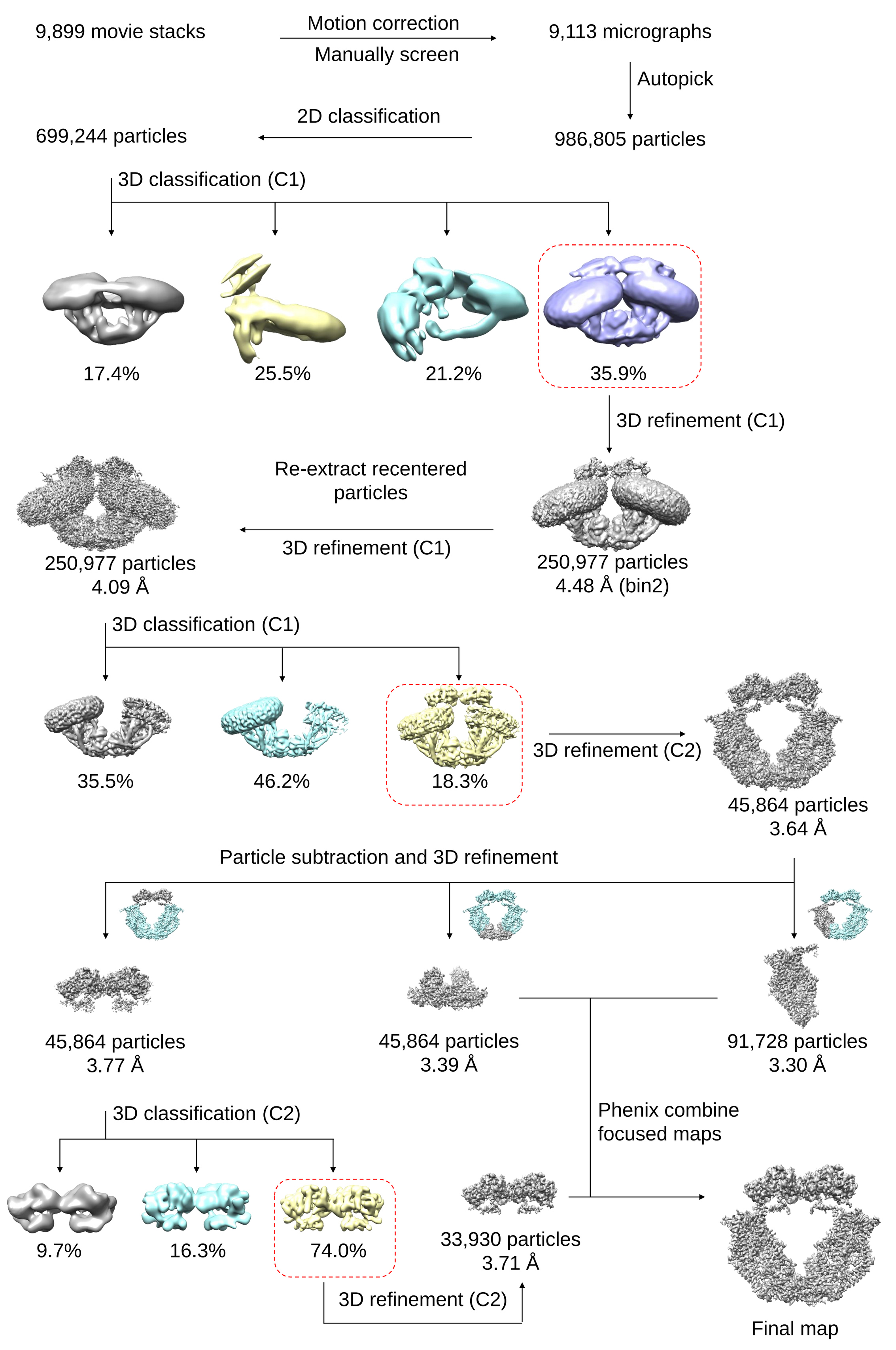
9,899 movie stacks
Motion correction
Manually screen
9,113 micrographs
Autopick
2D classification
699,244 particles
986,805 particles
3D classification (C1)
17.4%
25.5%
21.2%
35.9%
3D refinement (C1)
Re-extract recentered particles
250,977 particles
4.09 Å
3D refinement (C1)
250,977 particles
4.48 Å (bin2)
3D classification (C1)
35.5%
46.2%
18.3%
3D refinement (C2)
45,864 particles
3.64 Å
Particle subtraction and 3D refinement
45,864 particles
3.77 Å
45,864 particles
3.39 Å
91,728 particles
3.30 Å
3D classification (C2)
Phenix combine focused maps
9.7%
16.3%
74.0%
33,930 particles
3.71 Å
3D refinement (C2)
Final map

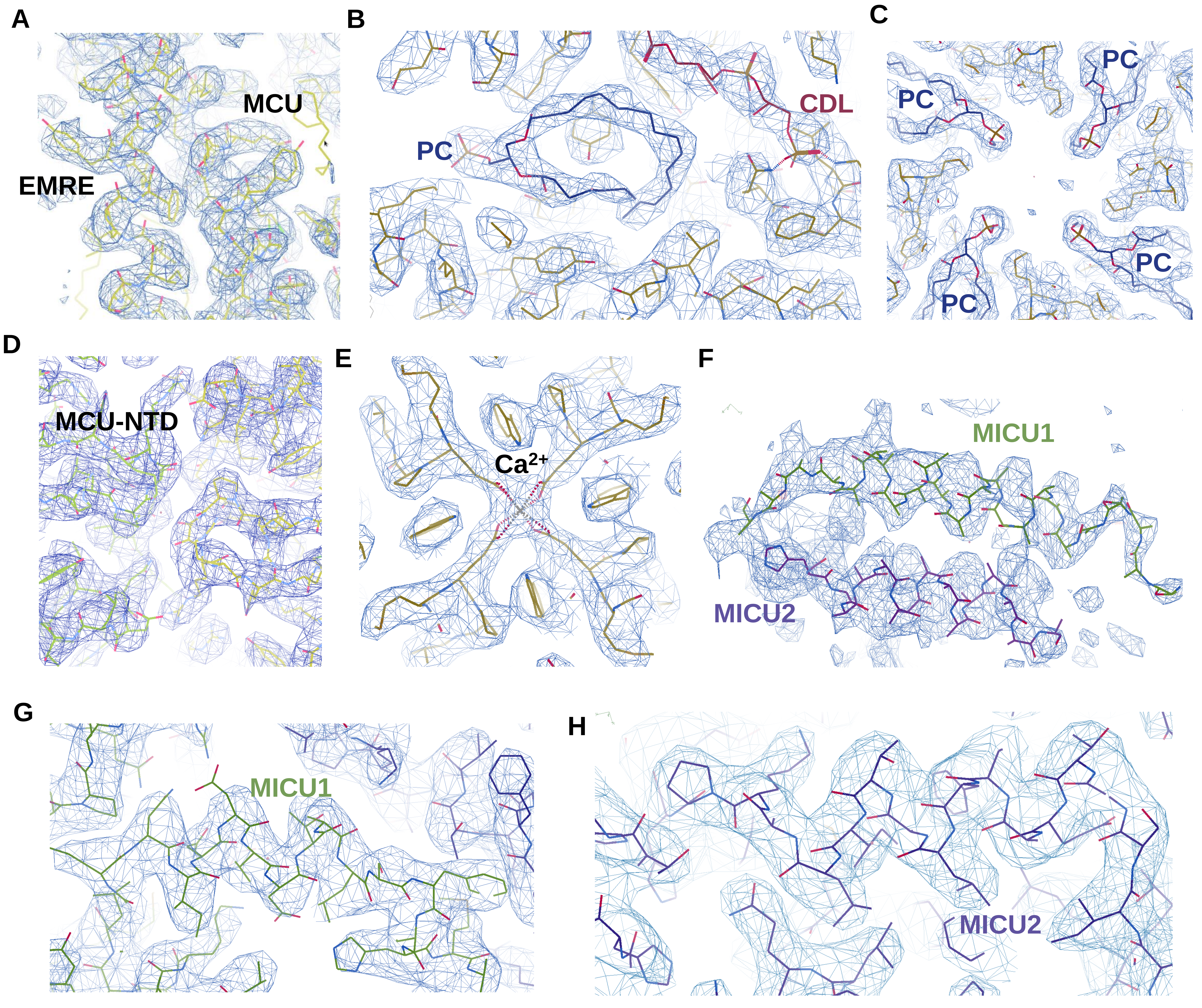

A
MCU
EMRE
B
PC
CDL
C
PC
PC
PC
PC
D
MCU-NTD
E
Ca2+
F
MICU1
MICU2
G
MICU1
H
MICU2

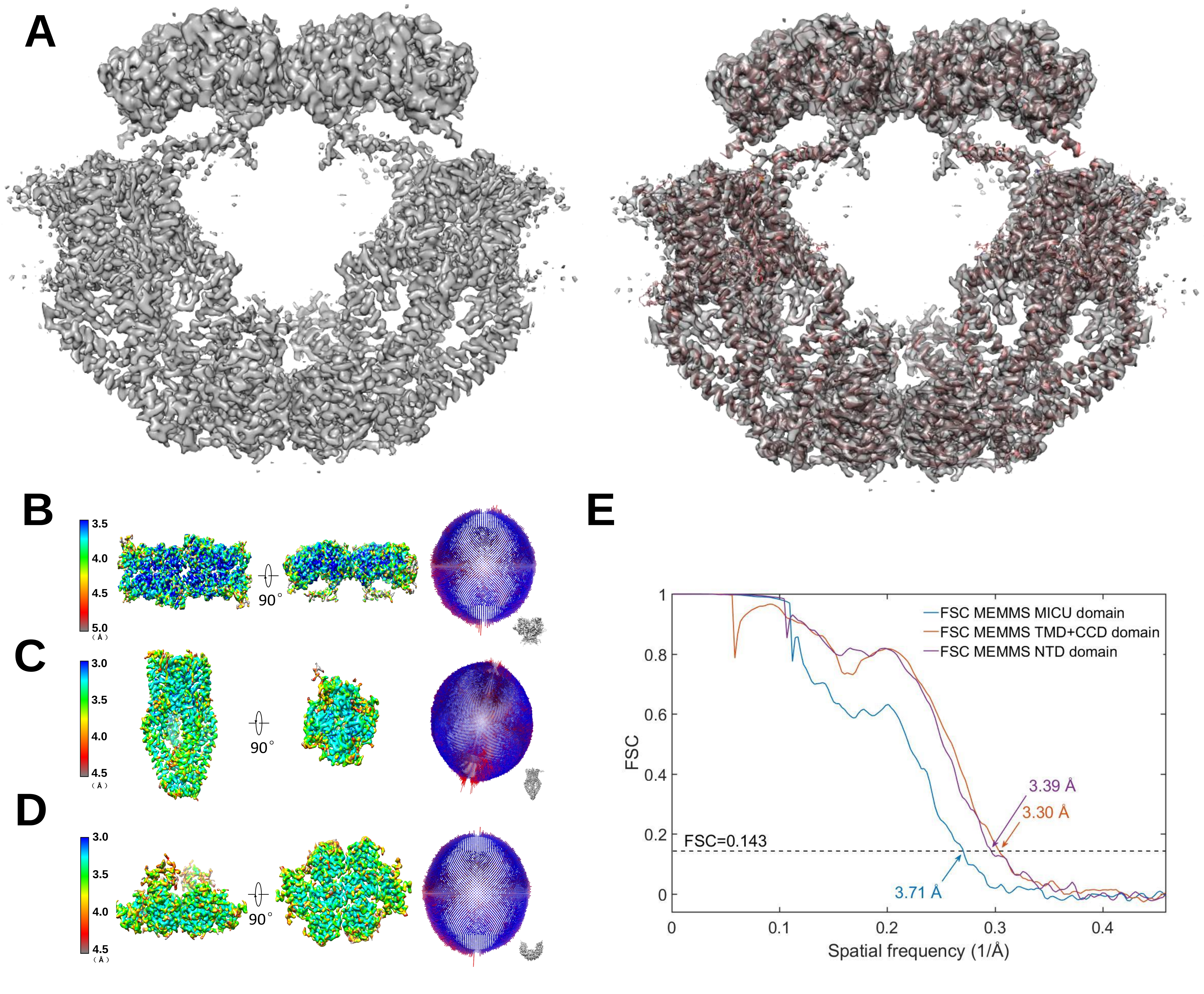
A
B
C
D
E
3.5
4.0
4.5
5.0
3.0
90°
FSC MEMMS MICU domain
FSC MEMMS TMD+CCD domain
FSC MEMMS NTD domain
FSC
FSC=0.143
3.39 Å
3.30 Å
3.71 Å
Spatial frequency (1/Å)

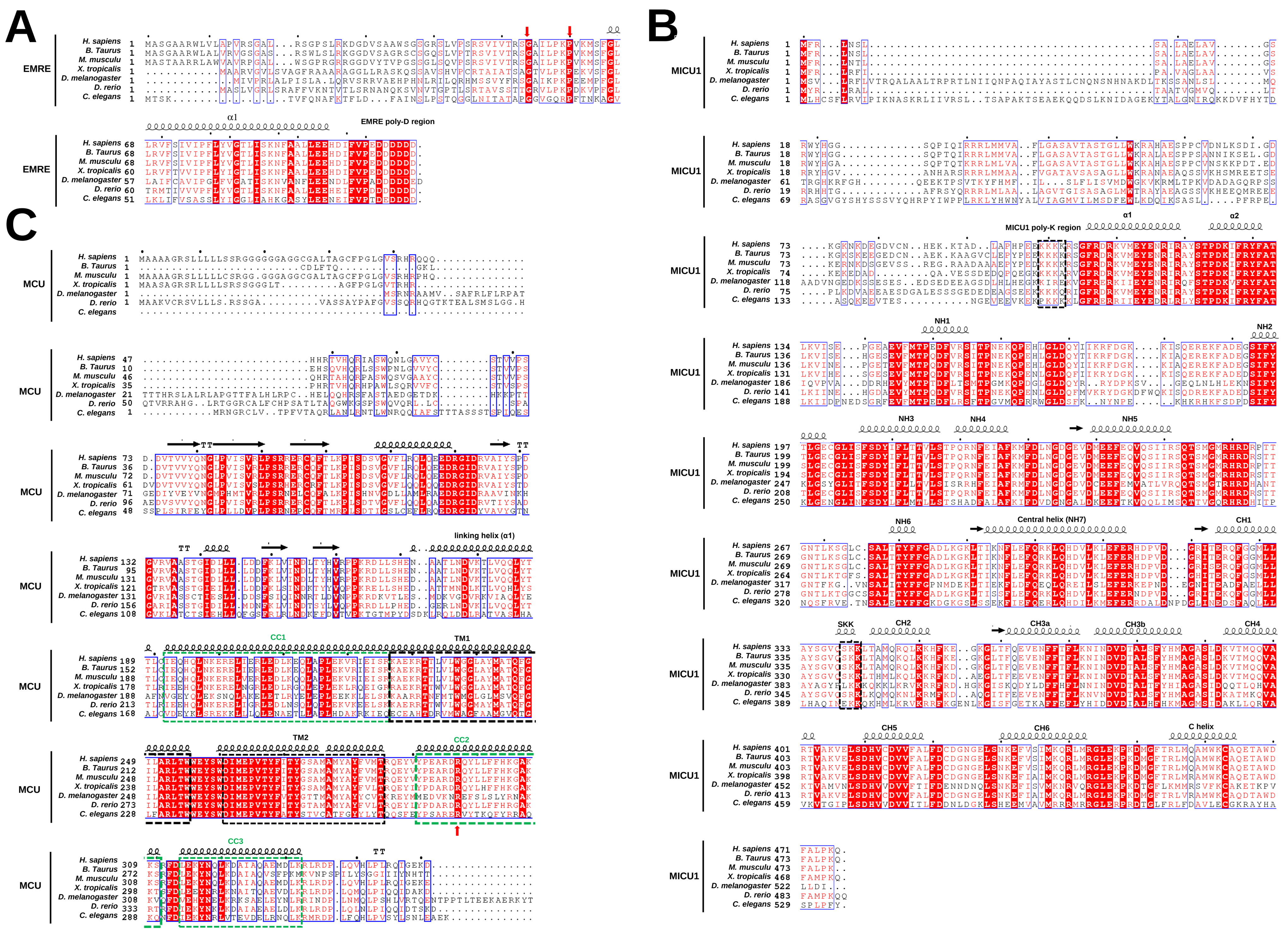
A
B
C
EMRE
MICU1
MCU
H. sapiens
B. Taurus
M. musculu
X. tropicalis
D. melanogaster
D. rerio
C. elegans
EMRE poly-D region
MICU1 poly-K region
linking helix (α1)
CC1
CC2
TM1
TM2
NH1
NH2
NH3
NH4
NH5
NH6
Central helix (NH7)
CH1
CH2
CH3a
CH3b
CH4
CH5
CH6
C helix
SKK

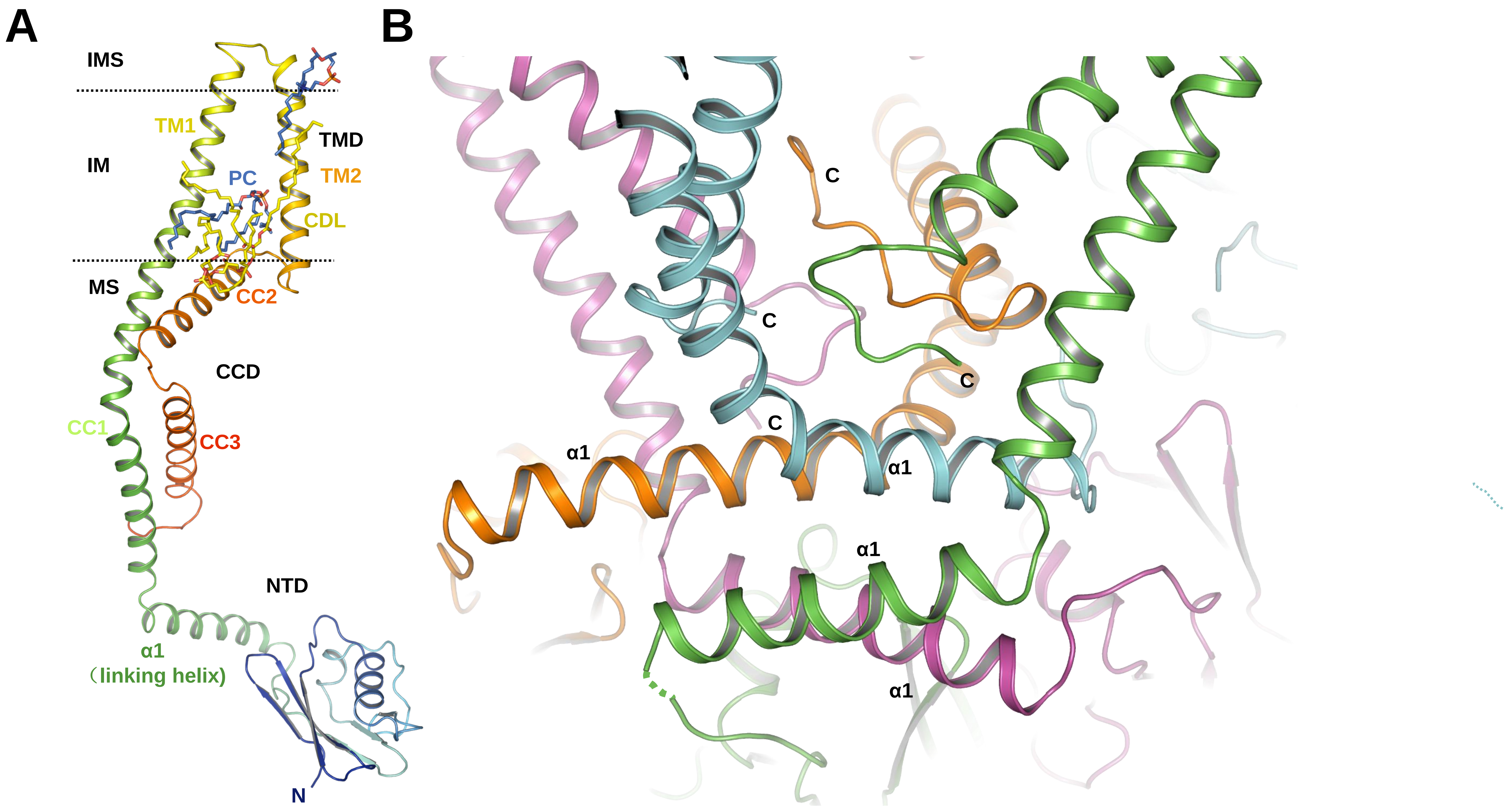
A
IMS
IM
MS
TM1
TMD
TM2
PC
CDL
CC2
CCD
CC1
CC3
NTD
α1
(linking helix)
N
B
C
C
C
C
α1
α1
α1
α1

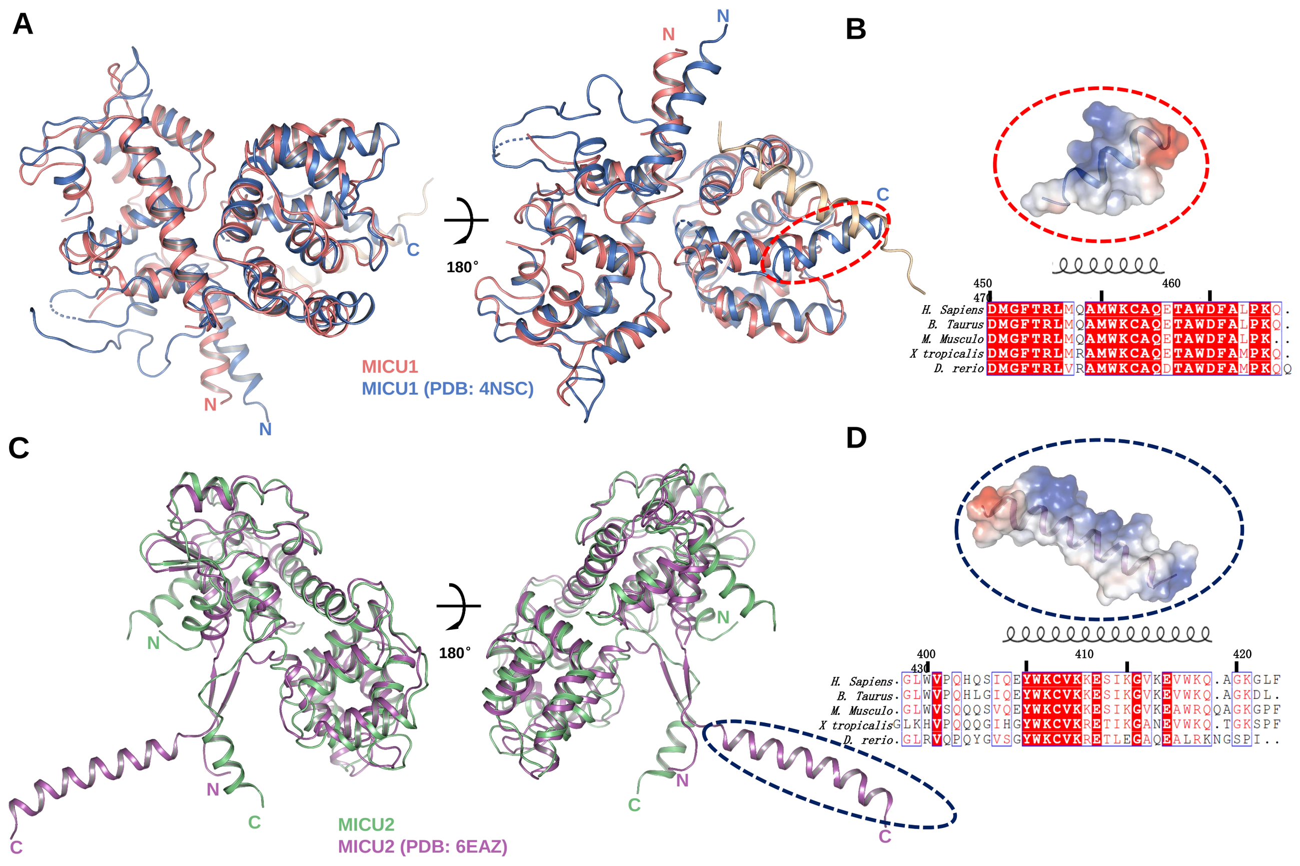
A
B
C
D
180°
N
C
MICU1
MICU1 (PDB: 4NSC)
MICU2
MICU2 (PDB: 6EAZ)

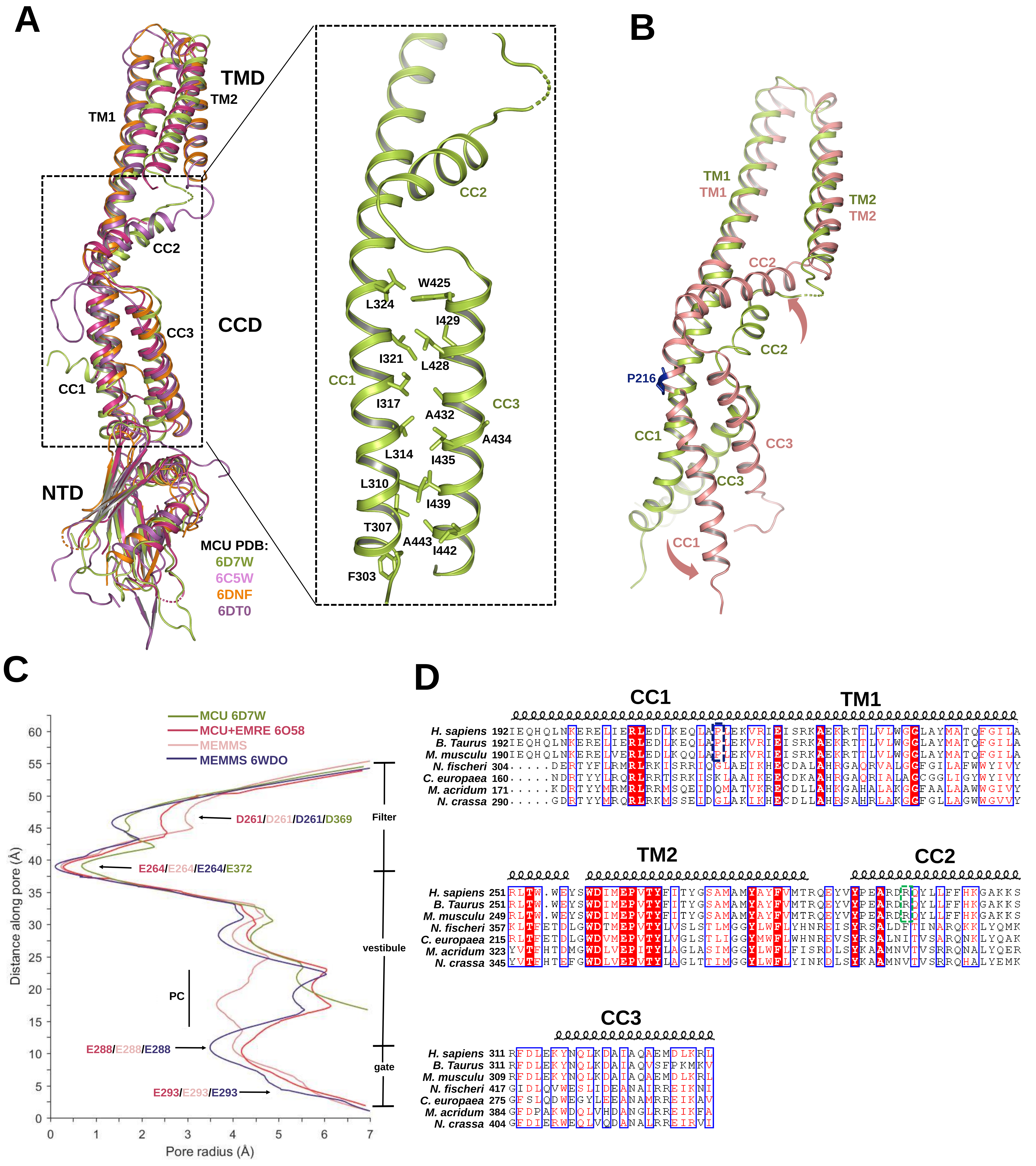

A
TMD
TM1
TM2
CC2
CC3
CCD
CC1
NTD
MCU PDB:
6D7W
6C5W
6DNF
6DT0
W425
L324
I429
I321
L428
CC1
I317
A432
CC3
A434
L314
I435
L310
I439
T307
A443
I442
F303
B
TM1
TM1
TM2
TM2
CC2
CC2
P216
CC1
CC3
CC3
CC1
C
MCU 6D7W
MCU+EMRE 6O58
MEMMS
MEMMS 6WDO
D261/D261/D261/D369
E264/E264/E264/E372
E288/E288/E288
E293/E293/E293
Filter
vestibule
gate
PC
Distance along pore (Å)
Pore radius (Å)
D
CC1
TM1
H. sapiens 192 IEQHQLNKERELIERRLEDLKEQLAPLEKVRIEISRKAEKRTTLVLWGGLAYMATQFGILA
B. Taurus 192 IEQHQLNKERELIERRLEDLKEQLAPLEKVRIEISRKAEKRTTLVLWGGLAYMATQFGILA
M. musculu 190 IEQHQLNKERELVERRLEDLKQQLAPLEKVRIEISRKAEKRTTLVLWGGLAYMATQFGILA
N. fischeri 304 ....DERTYFLRMRLRKISRRIQGLAEIKHECDALAHRGAQRVALGGFGILAFWWYIVY
C. europaea 160 ....NDRTYYLRQRLRRTSRKISKLAAIKEECDKAAHRGAQRIALAGCGGLIGYWYIVY
M. acridum 171 ....KDRTYYMRMRLRRMSQEIDQMATVKRECDLLAHKGAHALAKGGFAALAAWWGIVY
N. crassa 290 ....GDRTYYMRQRLRKMSSEIDGLAKIKHECDLLAHRSAHRLAKGGFGLLAGWWGVVY
TM2
CC2
H. sapiens 251 RLTW.WEYSWDIMEPVTYFITYGSAMAMYAYFVMTRQEYVYPEARDRQYLLFFHKGAKKS
B. Taurus 251 RLTW.WEYSWDIMEPVTYFITYGSAMAMYAYFVMTRQEYVYPEARDRQYLLFFHKGAKKS
M. musculu 249 RLTW.WEYSWDIMEPVTYFITYGSAMAMYAYFVMTRQEYVYPEARDRQYLLFFHKGAKKS
N. fischeri 357 KLTFETDLGWDTMEPVTYLVSLSTLMGGYLWFLYHNREISYRSALDFTINARQKKLYQMK
C. europaea 215 RLTFETDLGWDVMEPVTYLVGLSTLIGGYMWFLWHNREVSYRSALNITVSARQNKLYQAK
M. acridum 323 YVTFHTDMGWDLVEPITYLAGLASIMGGYLWFLFISRDLSYKAAMNVTVSRRQNALYQER
N. crassa 345 YVTFHTEFGWDLVEPVTYLAGLTTIMGGYLWFLYINKDLSYKAAMNVTVSRRQHALYEMK
CC3
H. sapiens 311 RFDLEKYNQLKDAIAQAEMDLKRL
B. Taurus 311 RFDLEKYNQLKDAIAQVSFPKMKV
M. musculu 309 RFDLEKYNQLKDAIAQAEMDLKRL
N. fischeri 417 GIDLQVWESLIDEANAIRREIKNI
C. europaea 275 GFSLQDWEGYLEEANAMRREIKAV
M. acridum 384 GFDPAKWDQLVHDANGLRREIKFA
N. crassa 404 GFDIERWEQLVQDANALRREIRVI
